# Pipeline Leakage Identification Based on Acoustic Sensors and EPSO-1D-CNN-Bi-LSTM Model

**DOI:** 10.3390/s25237355

**Published:** 2025-12-03

**Authors:** Niannian Wang, Kuankuan Zhang, Xingyi Wang, Bigang Peng, Shuwei Zhai

**Affiliations:** 1 Yellow River Laboratory, Zhengzhou University, Zhengzhou 450001, China; wnnian@zzu.edu.cn (N.W.); zkk_zzu@outlook.com (K.Z.); ymlzz@foxmail.com (B.P.); 2 National Local Joint Engineering Laboratory of Major Infrastructure Testing and Rehabilitation Technology, Zhengzhou 450001, China; 3 Collaborative Innovation Center of Water Conservancy and Transportation Infrastructure Safety, Zhengzhou 450001, China; 4 Henan Transport Investment Testing and Certification Co., Ltd., Zhengzhou 450121, China; 5 Henan Transportation Investment Group Co., Ltd., Sanmenxia Branch, Zhengzhou 450003, China; 17513215621@163.com

**Keywords:** water supply pipe, leakage detection, time domain analysis, multi-feature fusion, 1D-CNN-Bi-LSTM, EPSO

## Abstract

Water supply pipe systems are typically buried in the ground, leakage has always been a significant problem for urban water supply systems. Although leakage detection can be performed using in-pipe inspection devices with hydrophone modules, the accuracy is low and depends on staff experience, and long-term work can harm health. Therefore, leakage detection and classification of various leakage levels are crucial for pipelines. This study presents a one-dimensional convolutional neural network and bidirectional long short-term memory network fusion model (1D-CNN-Bi-LSTM) for leakage detection, with enhanced particle swarm optimization (EPSO) algorithm optimized hyperparameters and multi-feature fusion for data enhancement. Ablation experiments show the key roles of EPSO and Bi-LSTM modules, and full-scale experiments confirm the method’s effectiveness. Compared to other models, this one reaches 98.33% in both leakage detection and severity classification accuracy, with strong anti-noise ability and stable recognition. In conclusion, the proposed method reduces reliance on in pipe devices, offering a more accurate and effective solution for pipeline leakage detection and severity assessment.

## 1. Introduction

Urban water supply networks are essential infrastructures that ensure the reliable delivery of potable water to households, industries, and public services [1]. Pipeline leakage is a widespread issue that can cause significant water loss, environmental pollution, infrastructure deterioration, and safety risks [2]. Common causes of leakage include aging, corrosion, overloading, material degradation, and accidental damage [3]. Detecting leaks efficiently and accurately is therefore vital for maintaining the sustainability and safety of urban water networks.

From a broader perspective, leak detection is not only a technical challenge but also an essential operational measure for modern water supply enterprises [4]. Traditional sensing tools and manual inspections often fail to meet the requirements of real-time monitoring, especially in complex underground pipeline environments [2]. The existing methods encounter difficulties in handling various issues, including high environmental noise levels, limited effectiveness in low signal strength conditions, and difficulty in transferring laboratory results to actual environmental conditions [3,4,5]. These limitations highlight the need for more powerful, noise-resistant, and data-based detection strategies that can adapt to actual engineering scenarios. Based on these considerations, the main objective of this study is to develop a detection model that combines enhanced feature extraction with improved time series learning, utilizes advanced optimization strategies to achieve efficient parameter adjustment, and ultimately provides reliable leak identification under different operating and environmental conditions.

## 2. Literature Review

Traditional leakage detection techniques include manual inspection, artificial leak simulations, and a variety of advanced monitoring tools, such as the negative pressure wave (NPW) method, infrared thermography (IRT), ground-penetrating radar (GPR), transient hydraulic modeling, and acoustic detection [4,5,6,7,8,9,10,11,12]. For example, Li et al. applied the negative pressure wave method to denoised pipeline signals to locate leaks with short detection times and long monitoring distances, although this method requires the pipeline to remain airtight and stable [5,6,7,8]. Xie et al. combined IRT with convolutional neural networks (CNNs) to achieve automated leakage detection under complex noise environments [9]. Liu et al. demonstrated that ground-penetrating radar can effectively detect minor leakage by using diffraction-based two-dimensional (2D) and three-dimensional (3D) data interpretation [10]. Wu et al. proposed a leakage detection strategy based on transient hydraulic modeling [12], while Li et al. used acoustic detection to analyze leakage-induced vibration signals [13]. Among these approaches, acoustic detection is particularly advantageous due to its simple operation, high reliability, non-contact measurement capability, and wide applicability [14,15].

Despite these advances, several challenges remain in pipeline leakage detection. Real-world acoustic signals are often contaminated by internal noise—such as turbulence, pipe-wall scaling, and geometric irregularities—as well as external noise from vehicles, pedestrians, and environmental disturbances [16,17]. Many artificial intelligence–based studies rely on simulated or small-scale datasets, which limits their applicability to full-scale buried pipelines. Moreover, existing models rarely evaluate performance under low signal-to-noise ratio (SNR) conditions, even though this is critical for practical field testing. Although methods such as two-dimensional convolutional neural networks, one-dimensional convolutional neural networks, convolutional neural network–support vector machines, and convolutional neural network–long short-term memory hybrids have demonstrated promising results [16,17,18,19,20,21,22], the integration of advanced particle swarm optimization for robust parameter tuning and feature extraction remains insufficiently explored.

To address these gaps, this study develops an enhanced particle swarm optimization (EPSO)–based one-dimensional convolutional neural network and bidirectional long short-term memory network model (EPSO-1D-CNN-BiLSTM) for water pipeline leakage detection. Leakage signals are collected from full-scale buried pipelines under controlled pressure and aperture conditions. The model combines the feature extraction capability of the one-dimensional convolutional neural network with the temporal modeling ability of the bidirectional long short-term memory network. The Enhanced Particle Swarm Optimization algorithm introduces dynamic adjustment of inertia weight and learning factors, an early-stopping mechanism, and position restrictions to improve parameter optimization and classification accuracy. The proposed model is further evaluated under various signal-to-noise ratio conditions and leakage levels to verify its robustness and practical applicability.

Artificial intelligence and signal processing techniques have been widely applied in pipeline leakage detection. Two-dimensional convolutional neural networks have been used to classify spatial leakage patterns [16,17,18], whereas one-dimensional convolutional neural networks process raw time-series acoustic signals for real-time monitoring [19]. Hybrid approaches, such as convolutional neural network–support vector machines and convolutional neural network–long short-term memory models, combine spatial feature extraction with temporal learning to improve classification performance [20,21,22]. Optimization algorithms, including particle swarm optimization and its variants, have been incorporated to enhance hyperparameter tuning and feature selection [23,24,25,26]. More advanced techniques, such as the decreased and variable amplitude particle swarm optimization–least squares symmetric boundary support vector machine algorithm (DVAPSO-LSTSVM) and Gaussian mixture model (GMM)-based weighted least squares symmetric boundary support vector machine (G-WLSTSVM), further improve robustness under noise-dominated conditions [24,25]. However, gaps remain: performance under low signal-to-noise ratio conditions is rarely investigated; full-scale experimental validation is insufficient; and the joint optimization of one-dimensional convolutional neural networks and bidirectional long short-term memory networks using Enhanced Particle Swarm Optimization for extracting and learning leakage characteristics has not been thoroughly studied.

The main contributions of this paper include the following:

1. Conducting full-scale, on-site experiments to collect realistic leakage data from buried water supply pipelines under varying pressures and aperture sizes.
2. Developing an EPSO-1D-CNN-Bi-LSTM model to combine optimized feature extraction and bidirectional temporal sequence learning for improved leakage detection accuracy.
3. Incorporating EPSO mechanisms—including dynamic inertia weight adjustment, learning factor tuning, early stopping, and position restriction—to optimize 1D-CNN and Bi-LSTM parameter ratios.
4. Demonstrating the model’s robustness and effectiveness under different SNR conditions and leakage levels through extensive experimental validation.

The remaining sections of this paper are organized as follows: Section 3 describes the research and analysis methods, including the experimental design, signal acquisition, feature extraction, and model development. Section 4 presents the experimental results, the uncertainty analysis, and the planned future work. Section 5 provides the concluding remarks and outlines directions for future research.

## 3. Methodology and Analysis

### 3.1. Research Framework

**Figure 1:**
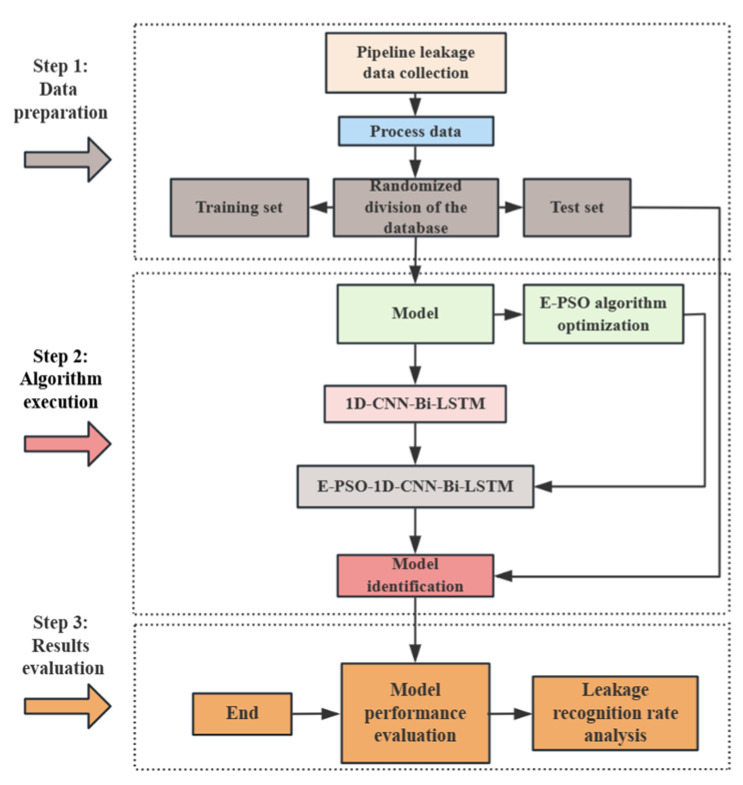
Overall Flowchart.

The research framework is shown in Figure 1. Firstly, The pipe-intrusion detection equipment with hydrophones (Snake-70S, Shenzhen Bomingwei Technology Co., Ltd., Shenzhen, China) was placed inside the full-scale pipeline to collect data under various operating conditions, such as normal data collected when the pipeline has no leakage (no leakage), collision between the equipment and the inner wall of the pipeline due to water flow impact (collision), different degrees of leakage near the pipeline leakage opening (minor leakage, moderate leakage, major leakage), and leakage data with different signal-to-noise ratios. Secondly, data processing is carried out, mainly including data cleaning of the signals to improve the quality and characteristics analysis of the experimental data. Finally, experimental verification is conducted, mainly involving the establishment of the EPSO-1D-CNN-Bi-LSTM model to identify and classify data of different working conditions and comparing the accuracy with other models. Through ablation experiments, the recognition and noise suppression performance of this model are verified.

### 3.2. Data Acquisition

The leakage of water supply pipelines mainly includes four types: pipe wall leakage, welding interface leakage, connection interface leakage, and pipe fracture leakage [16,27,28,29,30]. When a pipeline leaks, the hydraulic characteristics inside and outside the pipe will change significantly, resulting in abnormal pressure, flow, temperature, and flow rate in the leakage area. At the same time, due to the pressure difference between the inside and outside of the pipe, a jet will be generated at the leakage point, which will collide with the surrounding medium and generate an acoustic signal that can propagate along the pipe wall and the soil and gradually decay [31,32].

**Figure 2:**
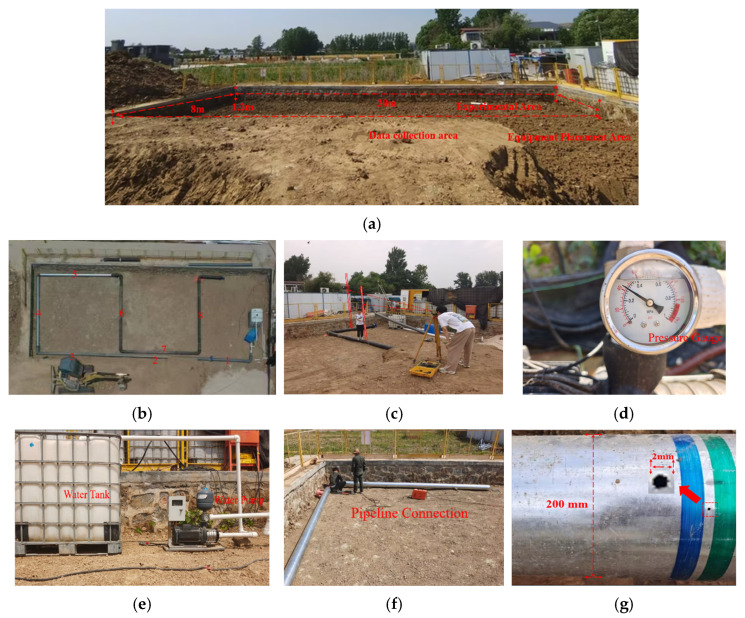
Experimental setup of the buried pipeline leakage simulation system. (**a**) Excavated test site. (**b**) Assembled DN200 pipeline system, where the numbers indicate the pipeline IDs. (**c**) Site leveling before installation. (**d**) Pipeline pressurization and water filling. (**e**) Water tank and water pump. (**f**) Pipeline construction and drilling operations. (**g**) Simulated leakage holes of different sizes.

To replicate realistic leakage conditions in buried water supply pipelines, a full-scale experimental site was constructed, as shown in Figure 2. The test pit (Figure 2a) measures 20 m in length, 8 m in width, and 1.2 m in depth, with a prepared base layer that prevents gravel and coarse sand intrusion. Prior to pipeline installation, the site surface was leveled using a precision leveling instrument (Figure 2c) to ensure that the DN200 pipeline system could be installed horizontally for stable equipment placement and accurate measurement. The pipeline system consisted of eight standard 6 m DN200 pipe sections, one 1 m section, and one 2 m section, forming a total length of 51 m with a burial depth of 1 m. These components were connected via flanges and adapters along with a constant-pressure water pump (equipped with a pressure gauge), a water tank, and two gate valves to assemble the complete water supply pipeline system, as illustrated in Figure 2b. After assembly, the system was fully pressurized and filled with water to ensure that no unintended leaks were present before soil backfilling and simulated leakage drilling.

Simulated leakage conditions were created by thinning the pipe wall and drilling holes at designated positions, with construction operations shown in Figure 2f. Following drilling, the upper portion of the pipeline was backfilled with the original soil and compacted three times using a mechanical tamper to reproduce real buried-pipeline conditions.

Two experimental schemes were designed to capture leakage data under varying severities. The first scheme maintained constant internal pressure while varying leakage aperture sizes. As shown in Figure 2g, a 2 mm hole was drilled in the second pipe section to simulate a small leakage condition, 4 mm holes were installed in the fourth and sixth sections for medium leakage, and a 6 mm hole in the eighth section represented a large leakage condition. The second scheme used a single leakage hole size while varying internal pressure levels. Based on readings from the pressure gauge (Figure 2d), pressures of 0.24 MPa, 0.30 MPa, and 0.36 MPa were applied to produce small, medium, and large leakage states, respectively. Through these two schemes, acoustic signals under multiple leakage severities were collected. Normal (non-leakage) data were recorded in areas without drilled holes; however, because leakage-induced vibrations decay significantly with distance along the pipe wall and soil, non-leakage data were collected at locations sufficiently far from all leakage points to avoid contamination.

**Figure 3:**
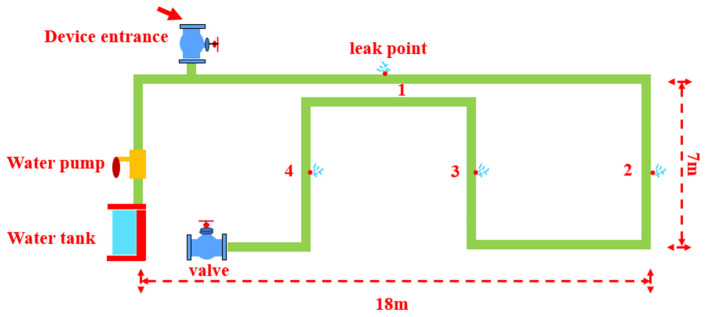
Experimental Layout and leakage point. The numbered labels in the diagram indicate the positions of individual leakage points.

**Figure 4:**
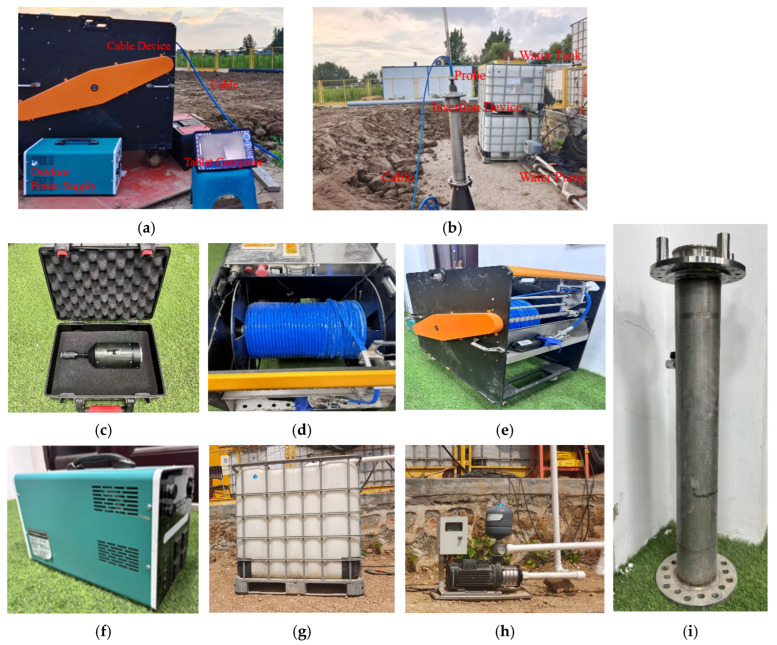
Detection equipment used in the full-scale experiment. (**a**) Internal pipeline detection device with hydrophone and control module. (**b**) Placement position of the detection equipment inside the pipeline. (**c**) A probe equipped with a hydrophone. (**d**) A cable car for facilitating the movement of equipment. (**e**) Cables used for data transmission. (**f**) Outdoor power supply. (**g**) Water tank. (**h**) Water pump. (**i**) The insertion device that helps probe enter.

As illustrated in Figure 3, this research uses an internal pipeline leakage detection device (Figure 4a) to enter the water supply pipeline through the gate valve opening (Figure 4b) to collect leakage signals (Figure 3). The device consists of a probe head (Figure 4c) responsible for collecting leakage data, including a hydrophone and control module, a cable car (Figure 4d) for facilitating the movement of the device, a cable (Figure 4e) for data transmission, an outdoor power supply (Figure 4f) with a maximum output power of 2500 W for outdoor use, a large-sized water tank (Figure 4g) for water supply, a water pump (Figure 4h) for generating the required internal pressure, a data receiving terminal tablet computer (Figure 4a), and an insertion device that helps the probe enter (Figure 4i).

The device is powered by the outdoor power supply (Figure 4f). The probe head (Figure 4c) is connected to the gate valve through the insertion device (Figure 4i) and sent into the pipeline. The movement of the probe head inside the pipe for data collection is controlled by the tablet computer terminal (Figure 4a) and the data acquisition was performed using the manufacturer-provided control software SnakeRobotControl (version V002F016D24102014, Shenzhen Bomingwei Technology Co., Ltd., Shenzhen, China). The collected data is transmitted back to the tablet computer terminal through the cable. It should be noted that the probe head (Figure 4c) supports a maximum sampling frequency of 24,000 Hz, a sensitivity of −210 dB, an accuracy of ±1 dB, and provides a stable frequency response between 10 Hz and 1000 Hz.

### 3.3. Data Preprocessing

During the actual leak detection work of the urban water supply pipeline network, various types of noise will interfere [33]. They can be mainly divided into internal noise sources within the pipeline and external noise sources outside the pipeline [31,32]. The internal noise sources mainly occur due to the increase in the service life of the pipeline, with severe pipe scale buildup, causing the local cross-sectional area of the pipeline to present an irregular shape. At the same time, the water flow area is greatly reduced, resulting in the unstable flow state of the water during its movement and generating noise. The external noise sources mainly include the sounds of vehicles traveling on the ground, the footsteps and speaking sounds of pedestrians, the operating sounds of large equipment, and so on.

**Figure 5:**
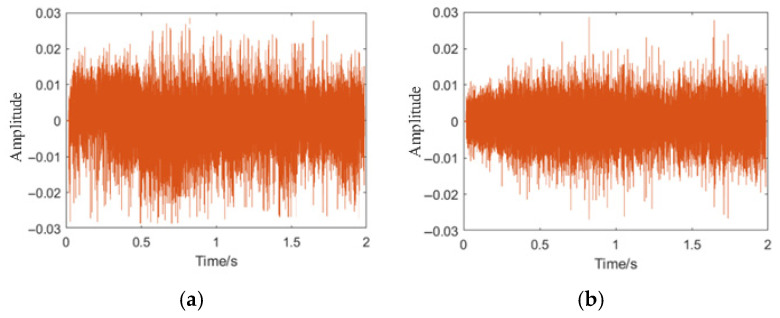

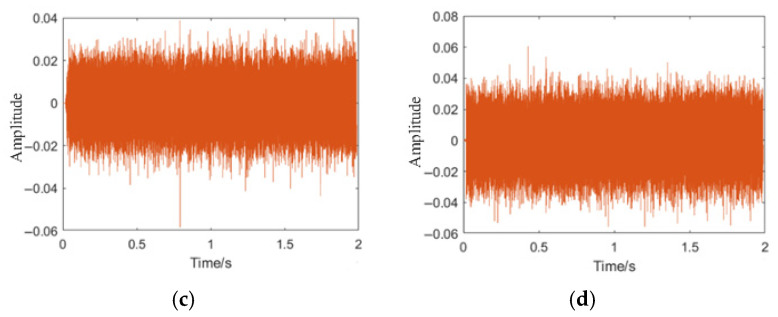
Collected leakage signals and processed time-domain waveforms. (**a**) Raw on-site leakage signal collected during the full-scale experiment. (**b**) Time-domain waveform of small-leakage signal after 6th-order Butterworth low-pass filtering. (**c**) Time-domain waveform of medium-leakage signal after filtering. (**d**) Time-domain waveform of large-leakage signal after filtering.

**Figure 6:**
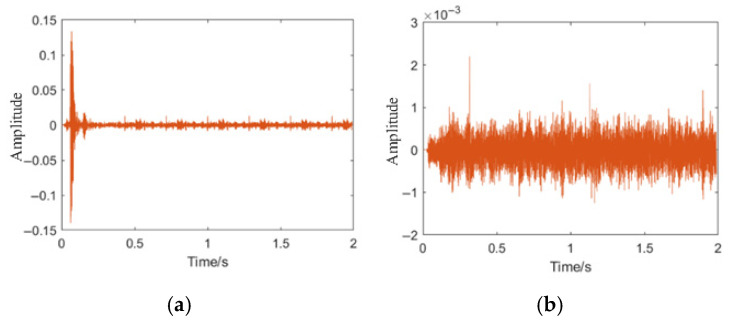
Time-domain waveforms of non-leakage and collision noise signals. (**a**) Time-domain waveform under external collision noise. (**b**) Time-domain waveform under non-leakage condition.

Due to the use of newly fabricated pipes, it is difficult to generate noise sources within the pipes. However, to further simulate the real collection situation, we added external noise sources (mainly including footsteps of pedestrians, speaking sounds, and engine operation of vehicles, etc.) outside the pipes when collecting data on the full-scale experimental field. Additionally, we separately collected environmental noise (including vehicle and crowd noise, etc.). To facilitate the control of the specific values of signal-to-noise ratio (SNR) for subsequent research and analysis, we also collected environmental noise separately. Here, we used data of five types of leakage, collision, no leakage, large leakage, medium leakage, and small leakage from the aforementioned collection pipes. Each type of data consists of 120 groups and is used for subsequent research and analysis. We resampled the collected data with a sampling rate of 8000 Hz and 16,000 sampling points, resulting in a time of 2 s. Figure 5a shows the on-site collected leakage signals, and Figure 5b–d are the time-domain graphs of small, medium, and large leakage signals after filtering and noise reduction through a 6th-order Butterworth low-pass filter. Figure 5b shows a set of time-domain graphs of the collected signals under small leakage. Figure 6a,b are the time-domain graphs when there is an external noise source collision and no leakage.

#### 3.3.1. Feature Analysis

Time-domain feature extraction is a fundamental step in signal analysis because it enables a direct evaluation of waveform characteristics such as vibration intensity, fluctuation patterns, and transient disturbances. In the context of water supply pipeline leakage, the acoustic signals originate primarily from three mechanisms: (1) friction between the escaping water and the pipe wall, which induces structural vibrations; (2) impact forces generated when high-pressure water jets collide with surrounding soil or other media; and (3) secondary collisions and friction among the surrounding materials driven by continuous high-pressure outflow. Although the specific physical processes differ, all leakage scenarios produce vibration-dominated acoustic signals, making time-domain analysis particularly suitable for identifying and characterizing leakage events.

Here, we extracted the time-domain characteristic values and entropy values, mainly including average value, kurtosis, pulse factor, and approximate entropy. As shown in Table 1, the average value is mainly used to represent the deviation degree of the vibration signal [34]. The kurtosis value (K) indicates the sharpness of the distribution shape of the leakage signal, to measure the thickness of the tail of the data distribution and the degree of convergence towards the center, to better distinguish the differences between signals under various working conditions [35]. The impulse Factor (I) mainly shows the proportion of the pulse component in the leakage signal. In this experiment, due to the different proportions of pulse components under different working conditions, it is also used as a characteristic value of the signal for subsequent analysis [36]. The approximate entropy (ApEn) measures the complexity of the leakage signal. When the leakage increases, the signal becomes more intense, and the complexity of the signal will increase, and its approximate entropy value will also increase, which is used to distinguish the signals under different working conditions [34,37].

#### 3.3.2. Leakage Detection Dataset

**Figure 7:**
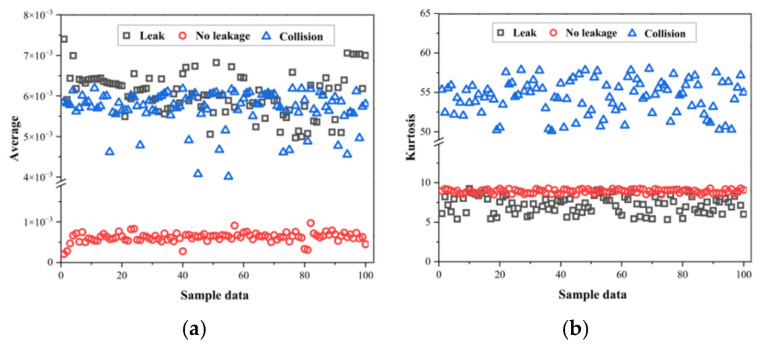

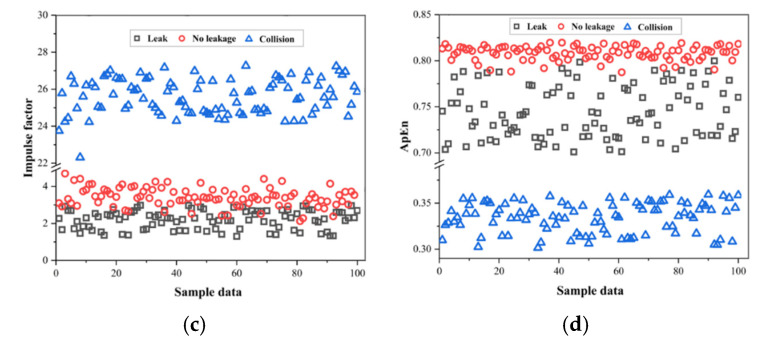
Extracted characteristic values under collision, normal, and leakage conditions. (**a**) Average value distribution for three operating conditions. (**b**) Kurtosis values for distinguishing collision and non-collision signals. (**c**) Impulse factor distribution under different operating conditions. (**d**) Approximate entropy values showing improved separability among the three conditions.

As shown in Figure 7, we extracted the characteristic values under three conditions: collision, normal, and leakage. Each condition consisted of 120 groups, totaling 360 datasets, which were used to test the performance of the established EPSO-1D-CNN-Bi-LSTM model in leakage detection.

In the case of no leakage, the signal inside the pipe only has weak noise with almost no fluctuations, which is consistent with the lowest extracted average characteristic value in Figure 7a and is significantly different from the other two signals. The average value of the leakage signal mainly falls within the range of 0.005 to 0.007. The average value of the friction and impact signal is concentrated between 0.004 and 0.006, and has a significant overlap with the average value of the leakage signal. This indicates that distinguishing these two signals based solely on the average value is challenging; therefore, we further considered the kurtosis under each condition, as shown in Figure 7b. Although kurtosis can separate the collision from the other two conditions, there are still overlapping parts between the leakage and non-leakage conditions. Next, we introduced the impulse factor as a characteristic value, as shown in Figure 7c, which has a similar effect to kurtosis and can only distinguish the differences between the collision condition and the other two conditions, but cannot clearly distinguish whether there is leakage or not; finally, we introduced approximate entropy, as shown in Figure 7d, which can better distinguish the three conditions compared to the previous three characteristic values, but there are still a small number of intersections between the non-leakage condition and the leakage condition. Therefore, we took all four of these as the input features of the subsequent model to increase the detection rate of leakage.

#### 3.3.3. Leakage of Classified Dataset

**Figure 8:**
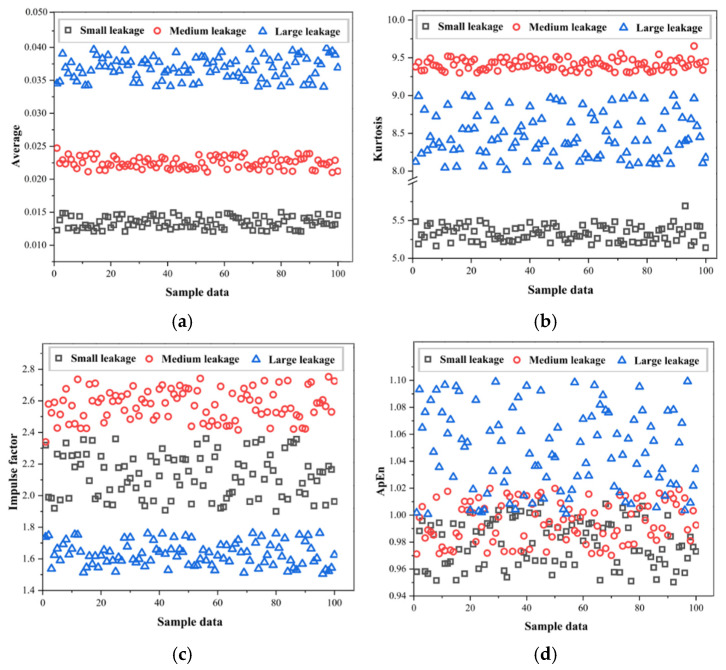
Extracted feature values under three leakage levels (high, medium, and low). (**a**) Average value distribution for different leakage levels. (**b**) Kurtosis values under high-, medium-, and low-leakage conditions. (**c**) Impulse factor distribution for the three leakage levels. (**d**) Approximate entropy showing improved separability among the leakage levels.

We have extracted the feature dataset used to test the performance of the EPSO-1D-CNN-Bi-LSTM model in identifying different levels of leakage. As shown in Figure 8, we have further extracted three types of leakage conditions (high, medium, and low) under the leakage conditions, with 120 groups for each type, totaling 360 data points, to test the model’s performance in classifying different levels of leakage.

After the above analysis, under this leakage condition, we still use the same feature values as in the previous section. As shown in Figure 8, since the intersection of the average value, kurtosis, and impulse factor features is very small, we will subsequently conduct ablation experiments to verify the impact of different feature combinations on the model performance.

### 3.4. Implemented Models

#### 3.4.1. 1D-Convolutional Neural Network (CNN)

The design of CNN involves convolutional computation and a deep structure, inspired by the biological receptive field mechanism [38,39].

**1.**  **Multi-Layer One-Dimensional Convolution**

The leakage data of pipeline acoustic signals mainly consists of time series data [33]. Time series typically possess characteristics such as temporal dependence, orderliness, coexistence of local and global features, noise sensitivity, high volume, and dynamic variation. Compared to traditional two-dimensional convolution, it is more suitable for processing one-dimensional sequence data and can fully utilize the temporal information of the sequence data. Therefore, we adopt a one-dimensional convolution layer and slide the convolution kernel along the time axis to extract the local features of the leakage signal.

We designed two one-dimensional convolutional layers. The first layer uses a convolution kernel with a size of filtersize = 6 and a total of round (pop (2)) convolution kernels. pop (2) mainly refers to the second element in the arrays popmin and popmax of the parameters of PSO, and the number of convolution kernels is determined through the optimization of PSO. The number of convolution kernels in the second layer is halved (filtersize/2), but the kernel size remains the same. This structure can gradually extract the features of the sequence data. The first layer captures low-level local features, and the second layer extracts higher-level features, enabling the network to learn more complex feature representations and improving the model’s ability to distinguish different types of leakage signals. The number of convolution kernels in the third layer is half of that of the second layer (round(pop (2)/4)), and the kernel size is the same as that of the second layer, extracting higher-level features. Additionally, a batch normalization layer and a rectified linear unit (ReLU) activation layer follow each convolutional layer. Batch normalization speeds up the training process and improves the model’s stability, while the ReLU activation function introduces nonlinearity, allowing the network to learn complex feature patterns and effectively enhancing the network performance.

**2.**  **Causal Convolution**

During the entire convolution process, using causal convolution (Padding = ‘causal’) ensures that the output of each time step only depends on the current and previous time steps, and is not influenced by information from future time steps. This is particularly important for tasks such as water pipeline leakage detection, as in practical application scenarios where future time step information is unavailable, causal convolution can guarantee that the model’s predictions conform to the causal relationship of the actual situation, that is, the collected pipeline leakage signal and the causal relationship between whether there is leakage and various leakage conditions, thereby improving the reliability and effectiveness of the model in water pipeline leakage detection.

**3.**  **Global Average Pooling**

**Figure 9:**
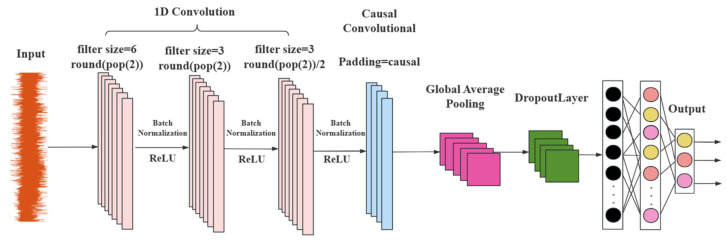
1D-CNN structure diagram.

At the end of the 1D-CNN section, a global average pooling layer (Global Average Pooling 1D Layer) was used to perform global average pooling on the data of each feature channel, compressing each feature channel into a scalar value. This not only reduces the dimensionality of the features, lowers the complexity of the model, but also retains the important information in the feature channels, while avoiding the overfitting problem that may occur when using fully connected layers. This makes the model more concise and efficient, and also provides a more compact feature representation for the subsequent Bi-LSTM layer. Figure 9 shows the structure diagram of the 1D-CNN model.

#### 3.4.2. Long Short-Term Memory (LSTM)

**Figure 10:**
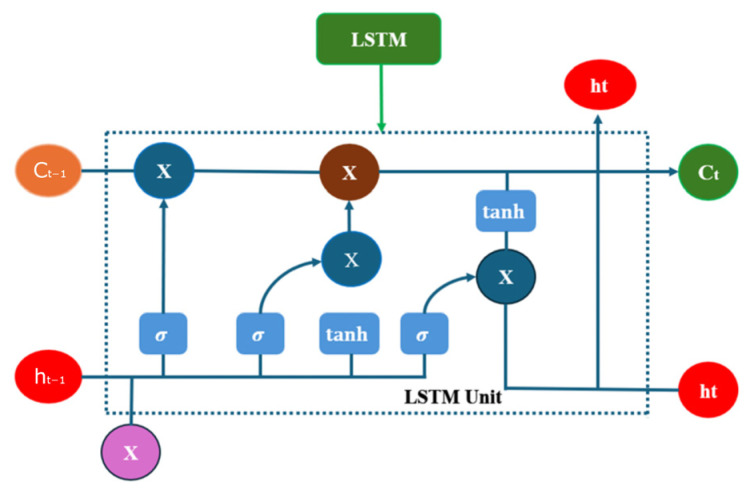
LSTM Diagram.

LSTM is a specialized RNN architecture designed to address the challenges of gradient vanishing and gradient explosion that traditional RNN encounter when dealing with long sequential data [40]. Figure 10 shows the unit diagram of LSTM.

**Figure 11:**
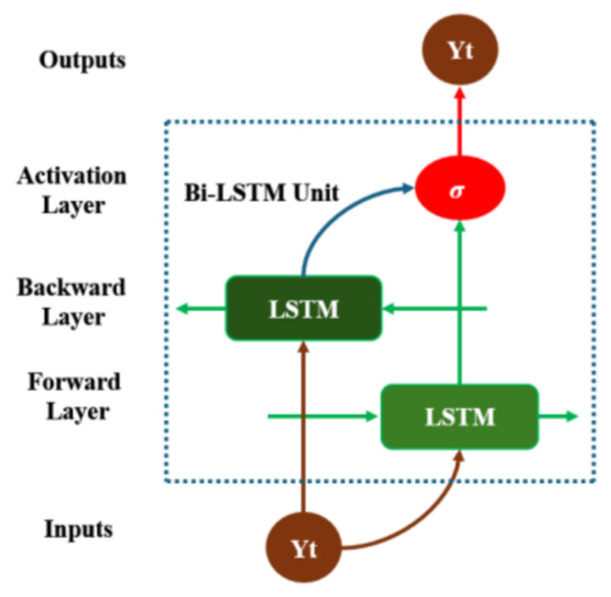
Bi-LSTM Diagram.

The bidirectional long short-term memory network (Bi-LSTM) is an improved recurrent neural network (RNN) specifically designed for processing and analyzing sequential data. Unlike the traditional unidirectional LSTM, Bi-LSTM incorporates two LSTM layers, one for the forward direction and one for the backward direction, within the network structure. This enables the model to simultaneously consider the past and future of the sequence information, enhancing its ability to understand contextual information, including the leakage information from before and after [41], as shown in Figure 11.

#### 3.4.3. Support Vector Machine (SVM)

Support Vector Machine is used to solve binary and multivariate classification problems. Its core idea is to classify samples of different classes by finding an optimal hyperplane in the feature space, which requires not only that it can separate two classes of samples, but also that the classification interval is maximized, i.e., the sample points closest to the hyperplane have the greatest distance to the hyperplane [42].

#### 3.4.4. 1D-CNN-Bi-LSTM

Although LSTM has been widely applied in fields such as time series prediction and natural language processing, its application in the specific domain of water pipeline leakage detection is relatively rare [41]. However, by integrating Bi-LSTM with 1D-CNN and introducing it into pipeline leakage detection, a new and effective solution has been provided for this field, which can more accurately identify and classify leakage signals and has practical application value. The Bi-LSTM layer receives the feature sequences extracted and processed by 1D-CNN as input, rather than directly processing the original data of pipeline leakage. Through the deep integration with 1D-CNN, Bi-LSTM can focus more on learning the long-term patterns and dynamic characteristics in the sequence data, improving the model’s ability to model complex sequence data. Finally, the Bi-LSTM layer is followed by a dropout layer (Dropout Layer = 0.4) and a fully connected layer (Fully Connected Layer, Dense = 3). The dropout layer is used to prevent overfitting by randomly discarding a certain proportion of neurons to enhance the model’s generalization ability. The fully connected layer maps the output of the Bi-LSTM layer to the output space of the classification task to improve the model’s performance.

The number of hidden units in the Bi-LSTM layer is dynamically determined by round (pop (3)), and pop (3) mainly refers to the third element in the arrays popmin and popmax of PSO in the following text. The number of hidden units is dynamically adjusted through the optimization of PSO, automatically optimizing the structure of the LSTM layer according to different levels of leakage datasets and improving the model’s performance and generalization ability.

**Figure 12:**
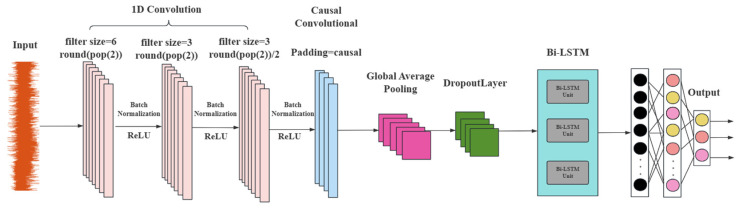
1DCNN-Bi-LSTM model structure diagram.

The 1D-CNN-Bi-LSTM model, as shown in Figure 12, combines the features of both 1D-CNN and Bi-LSTM architectures. Compared with the traditional network (recurrent neural network), Bi-LSTM has higher prediction performance in predicting time series data with long dimensionality and multivariate, it passes the extracted multivariate temporal features through memory gates such as forgetting gates, inputs, and outputs sequentially. Initially, the convolutional layer of 1D-CNN is employed to automatically extract features from the multi-featured variables of the leakage signal of the water supply pipe. Subsequently, the extracted features from the convolutional layer are transformed using the ReLU activation function. The nonlinear transformation of the activation function enhances the network’s ability to extract expressions. Ultimately, the Bi-LSTM transmits the extracted multivariate temporal features through memory gates, including forgetting gates, inputs, and outputs, in a sequential manner to categorize the leakage signals of varying types according to the extracted features.

#### 3.4.5. E-Particle Swarm Optimization (EPSO)

Particle Swarm Optimization is a method of optimization that mimics the foraging behavior seen in a group of birds [43]. E-Particle Swarm Optimization is an enhanced version of the Particle Swarm Optimization algorithm. We have introduced a dynamic adjustment mechanism for the inertia weight, which is different from the traditional static inertia weight. Instead, we adopt a strategy of dynamically adjusting the inertia weight. As the number of iterations increases, the inertia weight gradually decreases. This enables us to enhance the global search ability in the initial stage of calculation and is more conducive to local fine search in the later stage. We have also introduced an early stopping mechanism. During the iterative process, if the global optimal fitness value does not show significant improvement for several consecutive generations, it indicates that the algorithm may have converged or fallen into a local optimum. At this point, the algorithm can be prematurely terminated to avoid unnecessary calculations and save time. We have also introduced a position limitation mechanism, which is to limit the optimization range to improve the optimization effect of Particle Swarm Optimization. The specific optimization process is described in the next subsection.

#### 3.4.6. EPSO-1D-CNN-Bi-LSTM

When identifying pipeline leaks, the optimized EPSO-1D-CNN-Bi-LSTM model adopts a continuous iterative process. By establishing 1D-CNN and Bi-LSTM models suitable for pipeline leak detection, and then combining them, the EPSO algorithm is used to dynamically adjust the key parameters in the 1D-CNN-Bi-LSTM model to adapt to different leak datasets in the water supply pipeline. In this model, the prediction performance of different hyperparameter combinations varies. The key hyperparameters for the 1D-CNN-Bi-LSTM model used for pipeline leak classification and identification include learning rate, the number of convolutional kernels, and the number of hidden units (num_hidde_nunits), etc. The key hyperparameters of 1D-CNN-Bi-LSTM are optimized using the EPSO algorithm, which can find the ideal hyperparameter set to enable the model to achieve the highest recognition rate. The detailed process of enhancing the 1D-CNN-Bi-LSTM model using the particle swarm optimization algorithm is as follows:

1. Initialization: The initial solutions are randomly generated as X and V, representing the position and velocity of the birds in the flock, respectively. Each solution is associated with a specific hyperparameter of the model, including but not limited to num_estimators, learning_rate, num_filters, num_hidden_nunits, and so forth.

(1) $$X=\left[\begin{array}{cccc} {(x}_{1,1},u_{1,1}), & {(x}_{1,2},u_{1,2}), & \ldots , & {(x}_{1,d},u_{1,d})\\ {(x}_{2,1},u_{2,1}), & {(x}_{2,2},u_{2,2}), & \ldots , & {(x}_{2,d},u_{2,d})\\ \vdots & \vdots & \vdots & \vdots \\ {(x}_{i,1},u_{i,1}), & {(x}_{i,2},u_{i,2}), & \ldots , & {(x}_{i,d},u_{i,d}) \end{array}\right]$$

where i=1, 2…I represents the particle count, $(x_{i,d}$,$u_{i,d}$) indicates the position and velocity of the *i*th particle (i.e., a set of hyperparameter values), and D signifies the dimension.

2. Calculate the fitness: This investigation deals with a multi-classification problem, where the fitness function is defined as the model’s accuracy on the validation set.

(2) $$F=f(X_{i})$$

3. Particle velocity and position update: The formula for the *d*th-dimensional velocity update for the *i*th particle at each iteration is as follows:

(3) $$U_{id}^{k+1}=wU_{id}^{k}+c_{1}r_{1}\left(pbest_{id}^{k}-X_{id}^{k}\right)+c_{2}r_{2}\left(gbest_{id}^{k}-X_{id}^{k}\right)$$

(4) $$x_{i}^{k+1}=min\left\{popmax,max\left\{popmin,x_{i}^{k+1}\right\}\right\}\;\;\;$$

(5) $$w=w_{max}-\frac{w_{max}-w_{min}}{T_{max}}\times K$$

where w is the weight, $U_{id}^{k}$ is the velocity of particle i in the d-dimension at k iterations, $X_{id}^{k}$ is the current position of particle i in the d-dimension at k iterations, $pbest_{id}^{k}$ is the position (i.e., the coordinates) of the individual extremum of particle i, and $gbest_{id}^{k}$ is the position of the global extremum of the entire population, and $r_{1}$, $r_{2}$ are random numbers between [0, 1]. D stands for dimension, $c_{1}$, $c_{2}$ are acceleration coefficients (learning factors) that represent the weights of the statistical acceleration toward each particle’s push toward the pbest and gbest positions, and $X_{id}^{k+1}$ is the particle updating its position based on the updated velocity. w_max represents the initial inertia weight, w_min represents the minimum inertia weight, T_max represents the maximum number of iterations, and K represents the current iteration number. These are used to dynamically adjust the inertia weight. Popmax and popmin are, respectively, the lower and upper bounds of the position, ensuring that each dimension x does not exceed the allowable range. The value of the parameter in question determines whether particles will be able to remain in a state of suspension outside of the target region for a period before being pulled back, or whether they will be propelled with great suddenness towards or beyond the target region.

In the case where $c_{1}$ is equal to zero, the particle is devoid of cognitive capacity and is prone to converging on a local extremum as a result of particle interactions. Conversely, if $c_{2}$ is equal to zero, there is an absence of social information sharing among particles, and the algorithm turns into a randomized search with multiple starting points; if *c*1 = *c*2 = 0, the particles will keep on flying at the current speed until they reach the boundary. Usually *c*1, *c*2 is between [0, 4], here *c*1 = *c*2 = 2.5 is taken.

4. Update individual optimal position and global optimal position: Evaluate each particle’s current position fitness against its historical best, and update the optimal position if the current one is better, following these specific conditions:

(6) $$pbest_{id}^{k}=\left\{\begin{array}{c} X_{id}^{k},\;\;\;\;\;\;\;\;\;\;f(X_{id}^{k})<f(pbest_{id}^{k})\\ P_{id}^{k},\;\;\;\;\;\;\;\;\;\;f(X_{id}^{k})\geq f(pbest_{id}^{k}) \end{array}\right.$$

The global optimal position is chosen from the individual best positions of all particles based on the highest fitness:

(7) $$\;gbest_{id}^{k}=\arg min\;f\left(pbest_{id}^{k}\right)\;\;\;\;\;\;1\leq i\leq I$$

In this context, i represents the size of the particle swarm.

5. Whether to terminate: That is to say, define the maximum number of cycles or attain a certain level of fitness or other conditions as the criteria for stopping. Upon achieving a satisfactory end, deliver the global optimal position, meaning the best hyperparameter combination; otherwise, revert to step 3 to proceed with the iteration.

(8) $$\left[\begin{array}{cc} \mathrm{Optimal}\;\mathrm{parameter}\;\mathrm{combination},\;\;\;\;\;\; & n=N\\ \mathrm{Continuing}\;\mathrm{Iteration}, & n<N \end{array}\right]$$

In this context, n represents the number of iterations.

6. Moreover, to prevent the algorithm from getting stuck during the search process for an extended period, an early stopping mechanism is introduced under certain circumstances. The improvement of the global optimal fitness during the iterative process is recorded. If the improvement of the global optimal fitness is less than a certain threshold for several consecutive iterations (set as patience times), the early stopping will be triggered.

(9) $$|F_{minp}-F_{minp-1}|<tol$$

**Figure 13:**
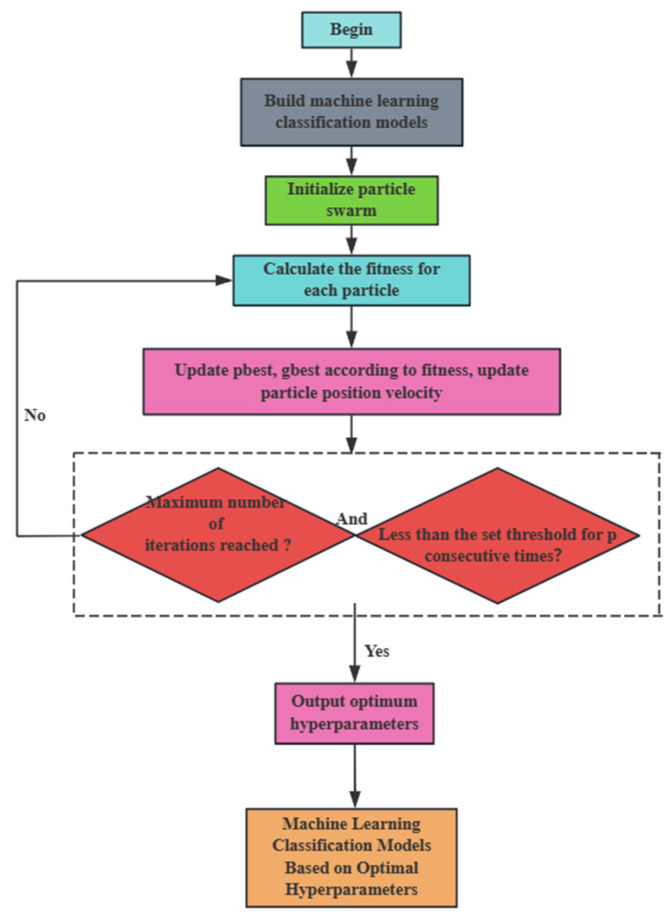
Flowchart of PSO-CNN-LSTM model.

Among them, F_mink represents the global optimal fitness of the *p*-th generation. As illustrated in Figure 13, the PSO-CNN-LSTM model is depicted as a flowchart. The optimal hyperparameter combination of this machine model is finally obtained after many iterations, after which the hyperparameter combination is utilized for model training and classification processing, and finally, the pipeline leakage and leakage degree are effectively identified.

### 3.5. Training Process

#### 3.5.1. Sample Data

Here we will be a variety of working conditions of 120 groups of a total of 360 groups of data. Table 2 and Table 3 shows the allocation of leakage data under different working conditions.

#### 3.5.2. E-PSO Hyperparameter Optimization

The selection of hyperparameters of the model is crucial for the model prediction results. The optimization models EPSO-1D-CNN-Bi-LSTM model and EPSO-SVM model are established by hyperparameter optimization. In the context of hyperparameter optimization, the EPSO is employed to navigate the search space, identifying the optimal hyperparameter configuration within the constrained range. Table 4 and Table 5 show the hyperparameters as well as the optimization range of each model, respectively. As illustrated in Table 6, the values for the hyperparameters of the EPSO model are presented. Table 7 shows the hyperparameter values after model optimization.

## 4. Results

### 4.1. Performance Metrics

In this study, we used Recall, Precision, F1 Score, FNR, and FPR as the key indicators for evaluating the performance of each model [2]. As shown in Table 8, Table 9, Table 10, Table 11, Table 12 and Table 13, the model optimized by EPSO demonstrated advantages in all the overall indicators. When the base model was replaced with SVM, the accuracy decreased and the F1 score also dropped, indicating that the 1D-CNN-Bi-LSTM model established in this study has significant advantages. Finally, a comparison was made with the DVAPSO-SVM model. Although the performance of the model improved significantly after multiple DVAPSO optimizations on SVM, it still did not have the accuracy and overall performance as good as the EPSO-1D-CNN-Bi-LSTM model established in this study.

(10) $$\mathrm{Precision}=\frac{\mathrm{TP}}{\mathrm{TP}+\mathrm{FP}}$$

(11) $$Recall=\frac{TP}{TP+FN}$$

(12) $$F1=2\times \frac{\mathrm{Precision}\times \mathrm{Recall}}{\mathrm{Precision}+\mathrm{Recall}}$$

(13) $$\mathrm{FNR}=\frac{FN}{TP+FN}$$

(14) $$\mathrm{FPR}=\frac{\mathrm{FP}}{\mathrm{TN}+\mathrm{FP}}$$

### 4.2. Comparison of Model Accuracy

**Figure 14:**
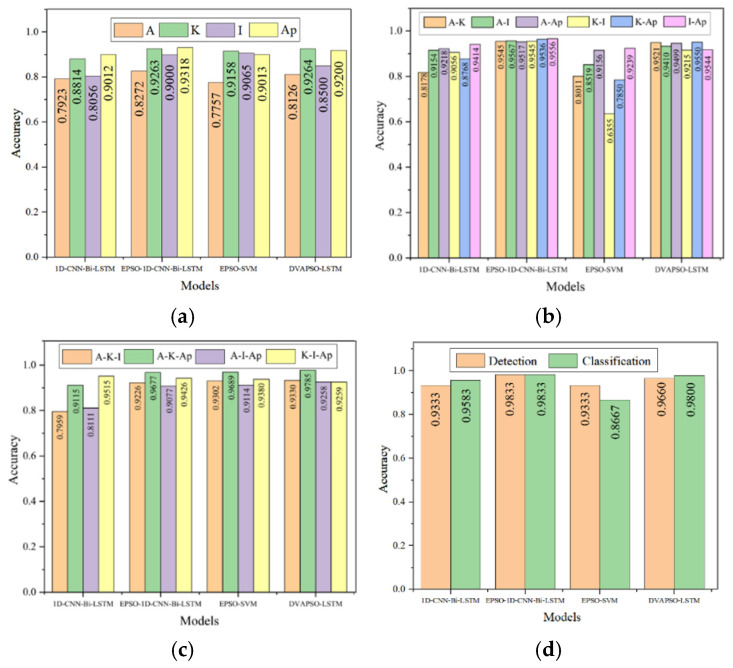
Ablation experiment results under different feature combinations. (**a**) Recognition results of leakage, no-leakage, and collision signals using the Impulse Factor (I) feature. (**b**) Recognition results using the Average–Kurtosis (A–K) feature set. (**c**) Recognition results using the Average–Kurtosis–Impulse Factor (A–K–I) feature set. (**d**) Recognition results using the full feature set (A–K–I–Ap), including classification performance for large-, medium-, and small-leakage levels.

We conducted ablation experiments, mainly analyzing the performance of the model after EPSO, the performance of the model after replacing the base model, and the influence of different features on the model performance, and finally compared it with DVAPSO-LSTM [22]. As shown in Figure 14, the recognition results of the model for three types of signals (leakage, no leakage, and collision) under different features are presented. Figure 14d contains the detection results of the three types of signals (leakage, no leakage, and collision) under four features, as well as the classification results for three leakage conditions (large, medium, and small). In the figure, Average, Kurtosis, Impulse Factor, and ApEn are represented by A, K, I, and Ap, respectively.

As shown in Figure 14, first, the leakage detection results under the same number of features were controlled. The number of control features was changed, and the recognition rate of the EPSO-1D-CNN-Bi-LSTM model after EPSO significantly improved. Figure 14a—The recognition rate under the pulse factor feature was the most significant improvement, from 80.56% to 90%; Figure 14b—The recognition rate under the Average-Kurtosis two features was the most significant improvement, from 81.78% to 95.45%; Figure 14c—The recognition rate under the Average-Kurtosis-Impulse Factor three features was the most significant improvement, from 79.59% to 92.26%; Figure 14d—The recognition rate under the Average-Kurtosis-Impulse Factor-ApEn four features was the most significant improvement, from 93.33% to 98.33%.

**Figure 15:**
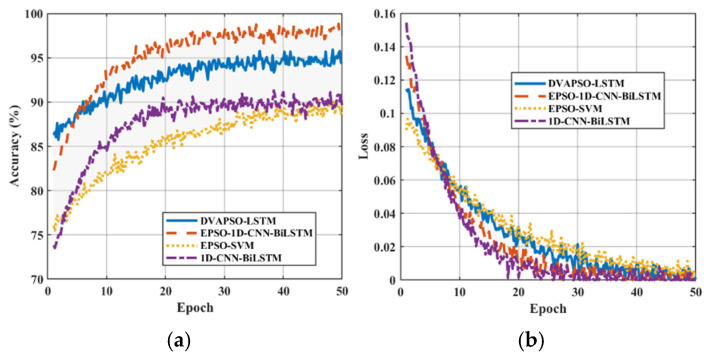
Performance comparison of different models. (**a**) Accuracy curves under different models. (**b**) Training and validation loss curves.

After replacing the base model, as shown in Figure 14, regardless of one feature or multiple features, the overall recognition rate of EPSO-SVM was generally much lower than that of the established EPSO-1D-CNN-Bi-LSTM model, proving the comprehensive advantages of the established model. Additionally, by increasing the number of features, the recognition rates of all models generally increased, indicating that the deep extraction of signal features is particularly important for all models. Figure 15 shows the accuracy curves and loss curves of different models.

In conclusion, after comparing each model with different features, different base models, different signal-to-noise ratios (SNR), and different evaluation indicators, the EPSO-1D-CNN-Bi-LSTM leakage model established in this study has obvious advantages.

### 4.3. Comparative Analysis of Anti-Noise Experiments

**Figure 16:**
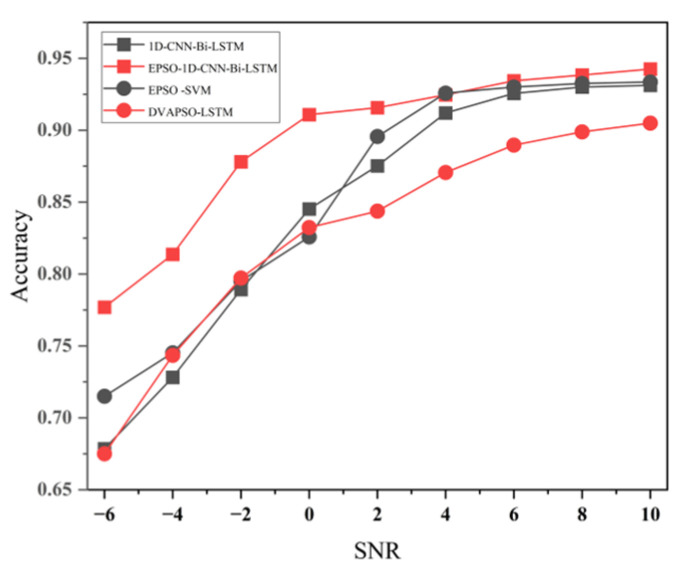
Recognition performance of different models under varying SNR conditions.

As shown in Figure 16, except for a few cases, the models established generally have a higher recognition rate than the DVAPSO-LSTM model. Additionally, by adding environmental noise to the signals as described in Section 3.1 to control the size of the signal-to-noise ratio (SNR) independent variable, the anti-noise performance of the models was verified.

We selected the leakage dataset from Section 3.1 and set the SNR to −6, −4, −2, 0, 2, 4, 6, 8, 10, etc. As can be seen from Figure 16, when the SNR is −6, more noise is added, and at this time, the recognition rates of all models have significantly decreased. However, the decline of other models is greater, and their anti-noise performance is worse. As the noise decreases, the recognition rates of each model gradually increase. When the SNR gradually increases to 10, the intensity of the noise is very small compared to the leakage signal intensity, and the recognition rates of all models almost stabilize and reach the peak. In summary, the EPSO-1D-CNN-LSTM model established in this study has the highest recognition rate and still maintains good performance in low SNR conditions, and has the best anti-noise performance.

### 4.4. Comparison of Different Optimization Algorithms

Among the various optimization algorithms, the Particle Swarm Optimization (PSO) algorithm was specifically selected for well-founded reasons. To verify the superiority of the PSO algorithm when combined with the CNN-LSTM model in this study, a series of comparative experiments were conducted against several conventional metaheuristic algorithms, including Falcon Hunting Optimization Algorithm (FHO), Coyote Optimization Algorithm (CO), Salp Swarm Algorithm (SSA), and Gray Wolf Optimizer (GWO) [44,45].

All algorithms were tested under identical experimental conditions, with the same maximum number of iterations (maxgen = 50), population size (sizepop = 5), optimization variables, and fitness functions. The dataset used for classification was the same as that collected in the previous experiments. Each experiment was repeated 25 times, and the number of cases achieving a recognition accuracy above 96% was used as the primary evaluation metric for assessing optimization performance.

**Figure 17:**
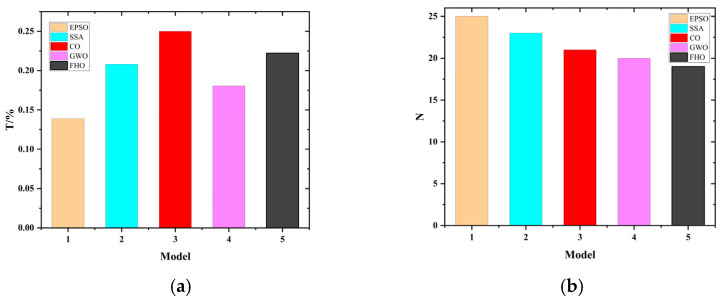
EPSO algorithm performance. (**a**) Classification time (T). (**b**) Number of classifications with accuracy > 96% (N).

As shown in Figure 17a, the time proportions for classification under different feature combinations demonstrate that the EPSO algorithm exhibits higher efficiency and the shortest processing time(T). Figure 17b presents the number of classifications achieving accuracy above 96%(N). The results indicate that the improved EPSO algorithm achieves the highest number of accurate classifications, demonstrating its superior stability and robustness compared with other optimization methods.

### 4.5. Discussion, Limitations, and Future Work

The proposed EPSO-1D-CNN-Bi-LSTM leakage detection model exhibits strong detection capability under low SNR conditions, as verified through full-scale buried pipeline experiments. The results demonstrate that the integration of CNN-based feature extraction, Bi-LSTM temporal modeling, and EPSO-driven hyperparameter optimization significantly enhances the model’s robustness and accuracy. Nevertheless, several important aspects merit further discussion.

Although real leakage signals were collected under controlled pressure and aperture conditions, the experiments still cannot fully replicate the variability encountered in long-term and large-scale water distribution networks. Factors such as heterogeneous soil environments, variations in pipe material, changes in burial depth, seasonal fluctuations in background noise, and long-distance attenuation may influence the acoustic characteristics of leakage signals. These uncontrolled environmental factors introduce inherent uncertainties into the experimental results, and their quantitative impact was not fully assessed in this study.

Future work will focus on conducting a systematic uncertainty analysis to quantify the reliability of the experimental results and model predictions. In addition, the dataset should be expanded to include more diverse pipeline conditions and environmental scenarios to improve model generalization. Developing lighter and more efficient network architectures will also be important for real-time deployment. Finally, integrating the model into an online monitoring system and validating its performance in long-term field applications will be key directions for future research.

## 5. Conclusions

In order to improve the accuracy of leakage detection in water supply pipelines, a high-precision leakage detection method under low signal-to-noise ratio conditions based on EPSO-1D-CNN-Bi-LSTM is proposed. This method first performs feature extraction of the signal, gradually establishes 1D-CNN, Bi-LSTM, and EPSO models, and finally establishes the EPSO-1D-CNN-Bi-LSTM leakage model to conduct leakage detection classification of the signal. The main conclusions are as follows:

1. Through on-site full-scale experiments, by controlling the pressure and the size of the aperture, real leakage data of buried water supply pipelines under actual conditions were collected, and the signal was analyzed for features.
2. By establishing the 1D-CNN-Bi-LSTM model and using the enhanced EPSO algorithm to optimize the hyperparameters of the model, the suitable PSO-1D-CNN-Bi-LSTM leakage model for pipeline leakage was finally established.
3. Through comparisons of different features, different basic models, different signal-to-noise ratios (SNR), and different evaluation indicators, the EPSO-1D-CNN-Bi-LSTM leakage model established in this study has obvious advantages compared to other models. The detection and classification accuracy can reach 98.33%, and it solves the problem of difficulty in detection under low SNR. And through comparative experiments, the stability and efficiency of the optimized model were proved.

## Figures and Tables

**Table 1:** Extracted Time Domain Features.

Signal Feature	Definition	Description
Mean value $\bar{Y}$	$\bar{X}=\frac{1}{N}\sum_{i=1}^{N}x_{i}$	Represents the overall amplitude level of the signal
Kurtosis K	K=$\frac{{E\left(X-\bar{X}\right)}^{4}}{\sigma^{4}}$	Measures the “peakedness” and impulsiveness of the signal amplitude distribution
Impulse factor I	$X_{\mathrm{peak}}=\underset{1\leq i\leq N}{max}|x_{i}|$ $I=\frac{X_{peak}}{\frac{1}{N}\sum_{i=1}^{N}\left|x_{i}\right|}$	Ratio of the signal’s peak value to its mean absolute value, reflecting the strength of transient impacts
Approximate entropy $ApEn\left(m,\;r,N\right)$	$X_{i}=\left\{x_{i},x_{i+1},\ldots ,x_{i+m-1}\right\}$ 1≤i≤N−m+1 $d(X_{i},X_{j})=\underset{0\leq k\leq m-1}{max}|x[i+k]-x[j+k]|$ $C_{i}^{m}(r)=\frac{\sum_{j=1}^{N-m+1}1\{d(X_{i},X_{j})\leq r\}}{N-m+1}$ $\Phi^{m}(r)=\frac{1}{N-m+1}\sum_{i=1}^{N-m+1}\;lnC_{i}^{m}(r)$	Measures complexity and irregularity of the signal; higher leakage intensity generally produces larger ApEn
	$ApEn\left(m,r,N\right)=\Phi^{m}(r)-\Phi^{m+1}(r)$	

**Note:** In the above table, $x_{i}$ represents the i-th sample of the signal, and N denotes the total number of samples. The symbol σ indicates the standard deviation of the signal, while $X_{\mathrm{peak}}$ is the maximum absolute amplitude of the signal, defined as $X_{\mathrm{peak}}={max}_{1\leq i\leq N}|x_{i}|$. For approximate entropy (ApEn), m denotes the embedding dimension, and r represents the tolerance or similarity threshold. $C_{i}^{m}(r)$ is the proportion of subsequences $X_{i}^{m}=\{x_{i},x_{i+1},\ldots ,x_{i+m-1}\}$ whose Chebyshev distance to other subsequences is less than r.

**Table 2:** Sample data allocation table.

Piping Conditions	No. of Training Sets	No. of Test Sets	Category Tags
Leakage	80	40	1
No Leakage	80	40	2
Collision	80	40	3

**Table 3:** Sample data allocation table.

Piping Conditions	No. of Training Sets	No. of Test Sets	Category Tags
Small leakage	80	40	1
Medium leakage	80	40	2
Large leakage	80	40	3

**Table 4:** A feature recognition result.

Hyperparameters	Optimization Range
learning_rate	Real (0.001,0.01)
num_estimators	Integer (100,1000)
num_filters num_hidde_nunits	Integer (12,64) Integer (5,60)

**Table 5:** SVM hyperparameters and optimization range.

Hyperparameters	Optimization Range
C	Real (0.001,0.01)
gamma	Integer (100,1000)

**Table 6:** Hyperparameter values for the PSO model.

PSO	Hyperparameter Value
C_1_	2.5
C_2_	2.5
maxgen	50
sizepop	5
(Vmax, Vmin)	(2,−2)
Optimize_num	4/2

**Table 7:** Hyperparameter values after model optimization.

EPSO	Hyperparameter Value
EPSO-1D-CNN -Bi-LSTM	learning_rate = 0.00248, num_estimators = 120, num_filters = 40, num_hidde_nunits = 41
EPSO-SVM	C = 10.16, gamma = 103

**Table 8:** Individual evaluation indicators of each model (Leakage).

Model	Recall	Precision	F1 Score	FNR	FPR	Category Tags
1D-CNN-Bi-LSTM	0.865	1	0.923	0.135	0	1 (Leakage)
EPSO-1D-CNN-Bi-LSTM	0.953	1	0.976	0.047	0	1 (Leakage)
EPSO-SVM	1	0.719	0.846	0	0.203	1 (Leakage)
DVAPSO-SVM	0.970	0.980	0.970	0.030	0.020	1 (Leakage)

**Table 9:** Individual evaluation indicators of each model (No Leakage).

Model	Recall	Precision	F1 Score	FNR	FPR	Category Tags
1D-CNN-Bi-LSTM	1	0.881	0.933	0	0.060	2 (No Leakage)
EPSO-1D-CNN-Bi-LSTM	1	0.951	0.975	0	0.025	2 (No Leakage)
EPSO-SVM	0.674	1	0.812	0.326	0	2 (No Leakage)
DVAPSO-SVM	0.980	0.980	0.980	0.020	0.020	2 (No Leakage)

**Table 10:** Individual evaluation indicators of each model (Collision).

Model	Recall	Precision	F1 Score	FNR	FPR	Category Tags
1D-CNN-Bi-LSTM	1	1	1	0	0	3 (Collision)
EPSO-1D-CNN-Bi-LSTM	1	1	1	0	0	3 (Collision)
EPSO-SVM	0.944	1	0.970	0.056	0	3 (Collision)
DVAPSO-SVM	1	1	1	0	0	3 (Collision)

**Table 11:** Individual evaluation indicators of each model (Small leakage).

Model	Recall	Precision	F1 Score	FNR	FPR	Category Tags
1D-CNN-Bi-LSTM	1	0.818	0.901	0	0.095	1 (Small leakage)
EPSO-1D-CNN-Bi-LSTM	1	0.952	0.975	0	0.025	1 (Small leakage)
EPSO-SVM	0.932	1	0.964	0.068	0	1 (Small leakage)
DVAPSO-SVM	0.950	0.96	0.975	0.050	0.040	1 (Small leakage)

**Table 12:** Individual evaluation indicators of each model (Medium leakage).

Model	Recall	Precision	F1 Score	FNR	FPR	Category Tags
1D-CNN-Bi-LSTM	0.838	1	0.911	0.162	0	2 (Medium leakage)
EPSO-1D-CNN-Bi-LSTM	0.950	1	0.974	0.05	0	2 (Medium leakage)
EPSO-SVM	1	0.957	0.978	0	0.026	2 (Medium leakage)
DVAPSO-SVM	0.960	0.960	0.975	0.040	0.030	2 (Medium leakage)

**Table 13:** Individual evaluation indicators of each model (Large leakage).

Model	Recall	Precision	F1 Score	FNR	FPR	Category Tags
1D-CNN-Bi-LSTM	0.957	1	0.977	0.043	0	3 (Large leakage)
EPSO-1D-CNN-Bi-LSTM	1	1	1	0	0	3 (Large leakage)
EPSO-SVM	1	0.970	0.985	0	0.011	3 (Large leakage)
DVAPSO-SVM	0.970	0.960	0.985	0.030	0.020	3 (Large leakage)

## Data Availability

The participants of this study did not give written consent for their data to be shared publicly, so due to the sensitive nature of the research supporting data is not available.
